# Antibiotics for the treatment of Cholera, *Shigella* and *Cryptosporidium* in children

**DOI:** 10.1186/1471-2458-13-S3-S10

**Published:** 2013-09-17

**Authors:** Jai K Das, Anum Ali, Rehana A Salam, Zulfiqar A Bhutta

**Affiliations:** 1 Division of Women & Child Health, The Aga Khan University, Karachi, Pakistan; 2 Global Child Health and Policy, Centre for Global Child Health, The Hospital for Sick Children, Toronto, ON, Canada

## Abstract

**Background:**

Diarrhea is a major contributor to the burden of morbidity and mortality in children; it accounts for a median of 11% of all deaths among children aged less than 5 years, amounting to approximately 0.8 million deaths per year. Currently there is a dearth of literature exploring the effectiveness of antibiotics for diarrhea due to Cholera, *Shigella* and cryptosporidiosis in children.

**Methods:**

We reviewed the literature reporting the effect of antibiotics for the treatment of diarrhea due to Cholera, *Shigella* and *Cryptosporidium* in children under five years. We used a standardized abstraction and grading format and performed meta-analyses to determine the effect of the treatment with various antibiotics on mortality and rates of clinical and bacteriological/parasitological failure. The CHERG Standard Rules were applied to determine the final effect of treatment with antibiotics on diarrhea morbidity and mortality.

**Results:**

For Cholera; the evidence was weak to recommend any effect on mortality. For *Shigella*; there was no data on mortality; either all-cause or cause specific, hence we used clinical failure rates as a proxy for *Shigella* deaths and propose that treatment of *Shigella* dysentery with antibiotics can result in a 82% reduction in diarrhea mortality due to *Shigella*. For cryptosporidiosis; there was data on all-cause mortality but the evidence was weak hence we used clinical failure rates as a proxy for mortality to estimate that antimicrobial treatment of diarrhea due to cryptosporidiosis can result in a 54% reduction in mortality.

**Conclusions:**

There is evidence to recommend antibiotic use for reduction of morbidity and mortality due to Cholera, *Shigella* and *Cryptosporidium*. We recommend that more clinical trials should be conducted to evaluate the efficacy and safety of first- and second- line drugs currently in use for treatment for diarrhea and dysentery in both developing and developed countries.

## Background

In 2011, 6.9 million children under five died, recording a decrease of 5.1 million from that of 1990 [1]. The progress is substantial but still a lot more is desired. Diarrhea is a major contributor to the burden of morbidity and mortality in children; it accounts for a median of 11% of all deaths among children aged less than 5 years, amounting to approximately 0.8 million deaths per year [1]. Most of this burden is concentrated in the developing countries of Asia and Africa.

An important cause of diarrhea in children in the developing world; Cholera is a rapidly dehydrating disease that can be fatal if untreated. Cholera was traditionally not considered a notable problem in young children; however data from endemic areas in the past two decades, suggests that young children, especially those aged less than 5 years are at the highest risk and bear the greatest burden of the disease [2-5].The burden of Cholera has been increasing steadily in the last decade and in the year 2010, the incidence of Cholera saw an increase of 130% from 2000, with an overall case fatality rate (CFR) of 2.38% and as high as 30% for some vulnerable groups living in high risk areas [6]. The World Health Organization (WHO) recommends rehydration as the mainstay of therapy for Cholera, antimicrobial therapy is advocated only in the management of severe cases-mostly interpreted as cases with “severe dehydration”; to reduce the severity of illness, shorten the duration of diarrhea and to reduce the duration of fecal excretion [7]. While the recommendations of International Centre for Diarrhoeal Disease Research, Bangladesh (ICDDR, B) do not restrict use of antibiotics to only “severe” cases and children with “some dehydration” who continue to pass large volumes of stool, are also candidates for antibiotic therapy [8,9].

Dysentery is a major cause of childhood morbidity and mortality [10] and a variety of pathogens are responsible for it including *Shigella*, *Salmonella*, *E. Coli* and *Campylobacter*. Of these, *Shigella* is responsible for most of the dysentery cases in the developing world [11]. The global incidence of *Shigella* is estimated at 80-165 million episodes annually, with 99% of episodes in the developing world [12]. A total of 69% of these episodes and 61% of all deaths attributable to shigellosis involve children under 5 years of age. According to previous estimates, 13.9% of infants and 9.4% of 1 to 4 year olds who are hospitalized with shigellosis die each year [13], while a recent review shows that shigellosis incidence is substantial and similar to the earlier estimate, but the updated death estimate is 98% lower [14]. The WHO recommends treating all cases of bloody diarrhea as suspected shigellosis and recommends treatment of shigellosis with ciprofloxacin and with three second-line antibiotics; pivmecillinam, azithromycin and ceftriaxone [10].

Cryptosporidium is a zoonotic intracellular protozoan parasite, which is an important cause of persistent diarrhea in children. It was included in the WHO Neglected Diseases Initiative in 2004. Cryptosporidium may cause life-threatening disease in people with AIDS and contributes significantly to morbidity among children in developing countries [15]. It may account for up to 20% of childhood diarrhea cases in developing countries and is a potentially fatal complication of AIDS [16]. Cryptosporidium infection in early childhood is also associated with poor cognitive function and failure to thrive [17]. Management of persistent infective diarrhea includes rehydration, adequate diet, micronutrient supplementation and antimicrobials [18]. Of established efficacy in immunocompetent patients, nitazoxanide is also useful for immunocompromised patients [19].

We reviewed the scientific evidence available for the use of antibiotics in the treatment of diarrhea due to Cholera, *Shigella* and *Cryptosporidium* in children, as well as differences in the effectiveness of various antibiotics. A Cochrane review [20] has evaluated the effectiveness of antibiotics for *Shigella* in children and adults, while the review by Traa et al [21] had evaluated the effectiveness of antibiotics for dysentery and estimated a cure rate of > 99%. We in this review have taken studies with confirmed cases of *Shigella *only, so have estimated the effect of antibiotics on *Shigella *cases. We have reviewed the available literature and evaluated the quality of included studies according to the Child Health Epidemiology Reference Group (CHERG) adaptation of Grading of Recommendations, Assessments, Development and Education (GRADE) criteria [22].

## Methods

We systematically reviewed literature published up to February 2012 to identify studies describing the effectiveness of antibiotics for the treatment of Cholera, *Shigella* and *Cryptosporidium* in children less than or equal to 5 years. Following CHERG Systematic Review Guidelines [22], we searched PubMed, Cochrane Libraries, Embase, and WHO Regional Databases to identify all published and unpublished clinical trials. Additional studies were identified by hand searching references from included studies.

We limited search to studies of antibiotic use in cases of acute diarrhea due to Cholera, *Shigella* and cryptosporidiosis. Search terms for Cholera included combinations of “Cholera”, “diarrhea”, “antibiotics” and “antimicrobial”, for *Shigella*, search terms included combinations of “shigell*”, “antibiotic”, “dysentery” and “antimicrobial” and for *Cryptosporidium*, search terms included combinations of “cryptosporidi*”, “treatment” and “antibiotic”. No language or date restrictions were applied in the search.

### Inclusion criteria

Studies were included if they reported the effect of antibiotics on morbidity and mortality associated with diarrhea due to the Cholera, *Shigella* and cryptosporidiosis in children, as observed by clinical and bacteriological failure and mortality. Only studies with a placebo group or no antibiotic control group were included for Cholera and *Cryptosporidium* diarrhea. For *Shigella*, there were no studies that compared antibiotics to placebo or a control group, so studies with an antibiotic comparison group were also included. Only studies with a confirmed diagnosis of the respective infection and on immuno-competent patients were included. Our original inclusion criteria included studies with children aged up to 5 years; however since only one study reported outcome for this age group, we expanded our inclusion criteria to include children up to sixteen years of age.

### Abstraction, analysis and summary measure

We abstracted data describing study identifiers and context, study design and limitations, intervention specifics and outcome effects into a standardized abstraction form for studies that met the final inclusion criteria as detailed in the CHERG Systematic Review Guidelines [22]. Clinical failure was defined as incomplete resolution or marked lack of improvement in signs and symptoms. Bacteriological/parasitological failure was defined as failure of clearance of the pathogen isolated from the patient on admission to the study, during or by the end of the treatment period.

Each study was assessed and graded according to the CHERG adaptation of the GRADE technique [22]. Randomized trials received an initial score of “high”. We deducted a grade point for each study design limitation. One- to two-point grade increases were allotted to studies with statistically significant strong levels of association (>80% reduction).

### Quantitative data synthesis

We conducted a meta-analysis for individual studies and pooled statistics were reported as the relative risk (RR) between the experimental and control groups with 95% confidence intervals (CI). Mantel–Haenszel pooled RR and corresponding 95% CI were reported or the DerSimonian–Laird pooled RR and corresponding 95% CI, where there was an unexplained heterogeneity. All analyses were conducted using the software Review Manager 5.1. Heterogeneity was quantified by Chi^2^ and I^2^, which can be interpreted as the percentage of the total variation between studies that is attributable to heterogeneity rather than to chance, a low p-value (less than 0.1) or a large chi-squared statistic relative to its degree of freedom and I^2^ values greater than 50% were taken as substantial and high heterogeneity. In situations of high heterogeneity, causes were explored by sensitivity analysis and random effect models were used.

We summarized the evidence by outcome, including qualitative assessments of study quality and quantitative measures, according to the standard guidelines. A grade of “high”, “moderate”, “low” and “very low” was used for grading the overall evidence indicating the strength of an effect on specific health outcome according to the CHERG Rules for Evidence Review [22].

## Results

### Cholera

**Figure 1:**
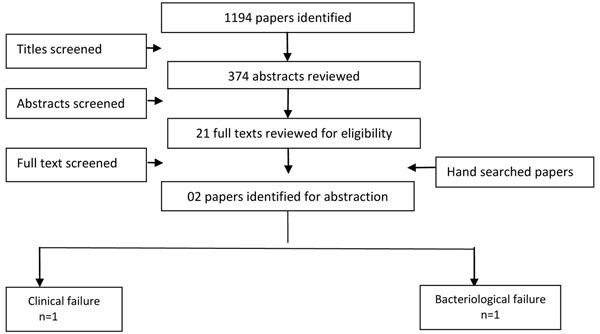
Search strategy flow chart for antibiotics treatment of cholera

We identified 374 titles from search conducted in all databases. After screening titles and abstracts, we reviewed 21 papers for the identified outcome measures of interest (Figure 1). Only one study reported data exclusively for children aged up to 5 years of age, so we expanded our study population to include children up to 16 years. Nine papers were reviewed and two [23,24] included in the final dataset as only these two studies had a suitable control or placebo group [23,24], whereas all other studies had comparison groups of different antibiotics. Both of the included studies were randomized control trials and were conducted in Bangladesh. One trial compared the antibiotics erythromycin, ampicillin and tetracycline, while the other compared erythromycin and trimethoprim/sulfamethoxazole TMP/SMX against a placebo group.

In Table 1, we report the quality assessment of studies by outcomes for antibiotic treatment of Cholera. Data for clinical failure was based upon findings of two data sets from one study [23]. There was a 63% reduction in clinical failure, with a RR of 0.37 (95% CI: 0.19, 0.71). Three datasets from one study were pooled for the outcome of bacteriological failure showing a significant 75% reduction with a RR of 0.25 (95% CI: 0.12, 0.53).

**Table 1:** Quality assessment of trials of antibiotics for the treatment of cholera

Quality Assessment						Summary of Findings		
				**Directness**		**No of events**		

**No of studies**	**Design**	**Limitations**	**Consistency**	**Generalizability to population of interest**	**Generalizability to intervention of interest**	**Intervention**	**Control**	**Relative Risk (95% CI)**

***Clinical Failure (Antibiotics vs. Placebo): Low Outcome Quality***								

01[23]	RCT	Only a single study		In developing country	Hospital based	8	20	**0.37 (0.19, 0.71)**

***Bacteriological Failure (Antibiotics vs. Placebo): Low Outcome Quality***								

01[23]	RCT	Only a single study		In developing country	Hospital based	6	22	**0.25 (0.12, 0.53)**

### Shigella

**Figure 2:**
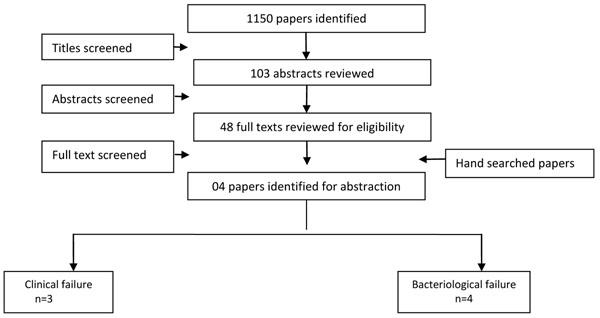
Search strategy flow chart for antibiotic treatment of *Shigella* dysentery

We identified 103 titles from the search conducted in all databases. After screening titles and abstracts, we reviewed 48 papers for the identified outcome measures of interest. Four papers [25-28] were included with data for multiple antibiotics and more than one outcome measure. We found three studies reporting clinical failure and four studies reporting bacteriological failure rates (Figure 2). All except one [29] were from the developing countries.

In Table 2, we report the quality assessment of studies by outcomes for antibiotic treatment of Shigella. Five datasets from three studies reported on the outcome of clinical failure. Two different antibiotics (pivmecillinam and ciprofloxacin) were used by these trials and it resulted in a 82% reduction in clinical failure with a RR of 0.18 (95% CI: 0.10, 0.33). While six datasets from four studies (pivmecillinam, ciprofloxacin and ceftriaxone) reported on bacteriological failure showing a reduction of 96% with a RR of 0.04 (95% CI: 0.01, 0.12).

**Table 2:** Quality assessment of trials of antibiotics for the treatment of Shigella

Quality Assessment						Summary of Findings		
				**Directness**		**No of events**		

**No of studies**	**Design**	**Limitations**	**Consistency**	**Generalizability to population of interest**	**Generalizability to intervention of interest**	**Intervention**	**Control**	**Relative Risk (95%CI)**

***Clinical Failure (Antibiotics): Moderate Outcome Quality***								

03[25-27]	RCT	No Control or placebo group, so used treatment failure rates	All suggest benefit	All studies were conducted in developing countries	Hospital based	50		**0.18 (0.1, 0.33)^b^**

***Bacteriological Failure (Antibiotics): Low Outcome Quality***								

04[25-28]	RCT	No Control or placebo group, so used treatment failure rates	All suggest benefit	All studies except one were conducted in developing countries	Hospital based	09		**0.04 (0.01, 0.12)^b^**

### Cryptosporidium

**Figure 3:**
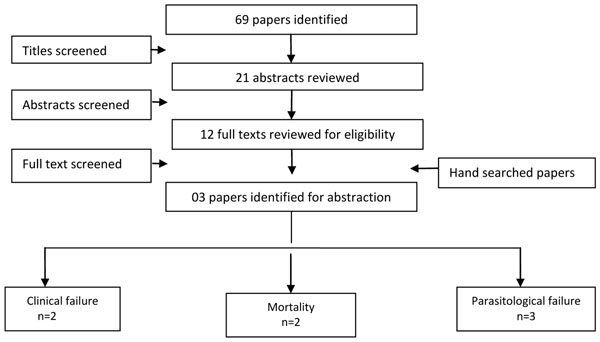
Search strategy flow chart for antibiotic treatment of *Cryptosporidium* diarrhea

We identified 1375 titles from the search conducted in all databases. After screening titles and abstracts, we reviewed 21 papers for the identified outcome measures of interest. Three [30-32] studies were included in the final dataset with most papers contributing data for more than one outcome measure (Figure 3). We found two studies reporting clinical failure rates and three studies reporting bacteriological failure rates and all-cause mortality was reported by two studies. All studies were conducted in developing countries and were randomized controlled trials.

In Table 3, we report the quality assessment of studies by outcomes for antibiotic treatment of cryptosporidiosis. Antibiotic (Nitazoxanide) treatment resulted in a significant 52% reduction in clinical failure rates with a RR of 0.48 (95% CI: 0.30, 0.75) as reported by two studies [30,31]. There was a 38% reduction in parasitological failure with a RR of 0.62 (95% CI: 0.46, 0.83) and was reported by three studies [30-32]. All-cause mortality was reported by two studies [30,32] and it shows that patients treated with antimicrobials (nitazoxanide and spiramycin) resulted in an insignificant 76% reduction in mortality with only seven events and a RR of 0.24 (95% CI: 0.04, 1.45).

**Table 3:** Quality assessment of trials of antibiotics for the treatment of cryptosporidium

Quality Assessment						Summary of Findings		
				**Directness**		**No of events**		

**No of studies**	**Design**	**Limitations**	**Consistency**	**Generalizability to population of interest**	**Generalizability to intervention of interest**	**Intervention**	**Control**	**Relative Risk (95%CI)**

***Mortality – All Cause (Antibiotics vs. Placebo): Low Outcome Quality***								

02[30,32]	RCT	One study used nitazoxanide while one used spiramycin	All studies suggested benefit	All studies were conducted in developing country	Hospital based	1	6	**0.24 [0.04, 1.45]^a^**

***Clinical Failure (Antibiotics vs. Placebo): Moderate Outcome Quality***								

02[30,31]	RCT		Both used nitazoxanide and suggested benefit	All studies were conducted in developing country	Hospital based	14	32	**0.48 (0.30, 0.75)^a^**

***Parasitological Failure (Antibiotics vs. Placebo): Moderate Outcome Quality***								

03[30-32]	RCT	Two studies used nitazoxanide While one used spiramycin	All studies suggest benefit	All studies were conducted in developing country	Hospital based	31	51	**0.62 [0.46, 0.83]^a^**

a= Fixed Effect Model

b= Random Effect Model

### Recommendation for the LiST model

**Figure 4:**
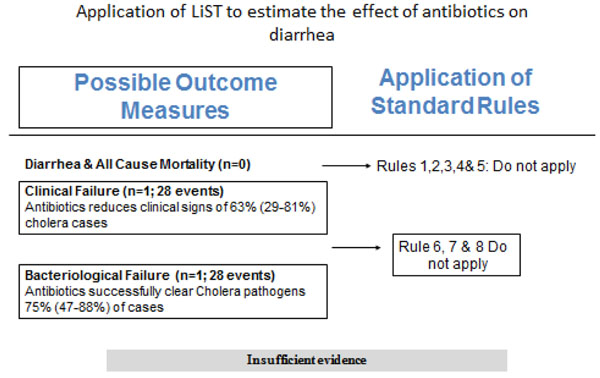
Application of standardized rules for choice of final outcome to estimate effect of antibiotics in diarrhea due to cholera

**Figure 5:**
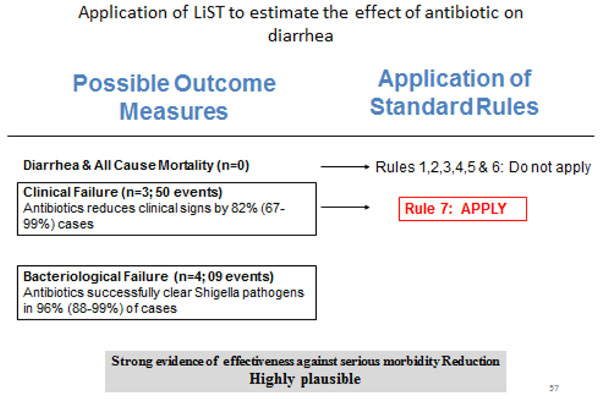
Application of standardized rules for choice of final outcome to estimate effect of antibiotics in *Shigella* dysentery

**Figure 6:**
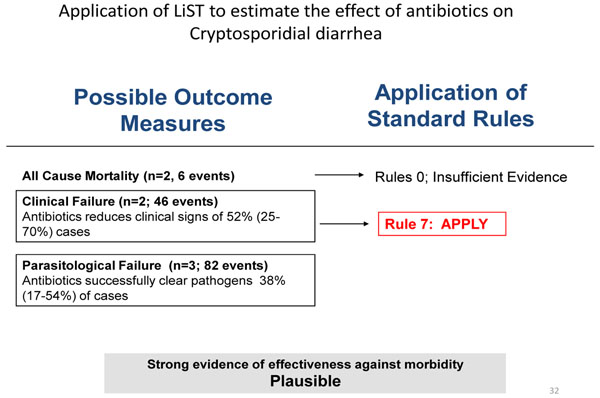
Application of standardized rules for choice of final outcome to estimate effect of antibiotics in diarrhea due to *Cryptosporidium*

Of the outcomes assessed for effect of antimicrobials on diarrhea in children, we applied the CHERG rules for evidence review to these outcomes. For Cholera, none of the outcomes had events greater than 50, so this was identified as insufficient evidence for LiST estimation (figure 4). For *Shigella*; there was no data on mortality; either all-cause or cause specific, hence we used clinical failure rates as a proxy for *Shigella* deaths and propose that treatment for *Shigella* dysentery with antibiotics results in an 82% reduction in diarrhea mortality due to *Shigella* (Figure 5). For cryptosporidiosis; there was data on all-cause mortality but the evidence was weak as there were only seven events hence we used clinical failure rates as a proxy for mortality to estimate that antimicrobial treatment of diarrhea due to cryptosporidiosis results in a 54% reduction in mortality (Figure 6).

## Discussion

This systematic review concludes that antibiotics when given for Cholera reduce the clinical and bacteriological failure rates; however the evidence for reducing morbidity in children is insufficient to recommend antibiotic use in all cases. It should also be noted that the studies included in our review are more than a decade old and hence conclusions drawn from these studies regarding the antibiotics used may not reflect the current changing patterns of antibiotic resistance among vibrio Cholerae strains worldwide. The antibiotics used for *Shigella* dysentery included ciprofloxacin, pivmecillinam and ceftriaxone and there is strong evidence that antibiotics significantly reduce clinical failure and bacteriological failure rates. It is evident that the use of antibiotics along with appropriate rehydration therapy can lead to significant reduction in morbidity and associated mortality. The antibiotics used for Cryptosporidium in our review were nitazoxanide and spiramycin and we conclude that antibiotics significantly reduced parasitological and clinical failure, however, all-cause mortality, while evaluated by two studies [30,32] reduced mortality by 76% but the effect was insignificant and based on a small sample size.

Diarrhea due to Cholera is an important cause of morbidity and mortality in children in the developing world [33]. The cornerstone for Cholera therapy remains prompt and adequate rehydration. Antibiotics are known to shorten the course of illness, decrease stool output, decrease the requirements of rehydration fluid and decrease infectivity of the patient [29,34,35]. It also leads to less home shedding and transmission to households [35] which in turn would lead to less nursing care and patients leaving the treatment center earlier. This approach would maximize the effectiveness in resource-limited settings while optimizing patient care. There are concerns that over emphasis on the use of antibiotics may lead to prescription in all cases of Cholera, but training and education can handle this. Although the evidence is weak as there are a few studies evaluated and more research is needed, we propose that antibiotics have a potential in moderate and severe Cholera and there have been no adverse events identified by any study.

Shigellosis is an acute and invasive infection that is commonly manifested as bloody diarrhea. Like Cholera, it remains an important cause of morbidity and mortality in children in the developing world. While rehydration and adequate nutrition are important, antibiotic therapy is the mainstay of treatment for *Shigella* dysentery which is known to hasten clinical recovery and reduce the likelihood of complications and death [10].

*Cryptosporidium* is responsible for persistent diarrhea in children, causing a major impact on child health [36]. Disease varies in children with intact immune system versus those with compromised immunity [37]. Initially thought to be a disease of the immune-compromised, immune-competent individuals can also acquire the infection with mild-to-severe diarrhea lasting from several days to weeks and our analysis has shown benefits of antibiotic therapy in immune competent patients.

The studies contributing data in this review were mostly conducted in developing countries hence increasing the generalizability of the studies to children in low and middle income countries with the highest diarrhea mortality rates. However considering the variations in antibiotic sensitivity among strains worldwide, our findings may not be generalizable to all populations. Almost all studies were conducted in a hospital setting where treatment was monitored by hospital staff. Thus it is possible that the therapeutic effect of antibiotics demonstrated by the meta-analyses may not be completely reproducible in a community or out-patient setting, due to poor compliance with administration of the right dosage at correct timings and for the prescribed time period. Also adequate rehydration and subsequent monitoring of the child for dehydration are extremely important and this may not be performed properly by the caretakers in outpatient or community setting, which may not give the desired results, despite antibiotic use. Diagnostic facilities are also not available in the community, so future recommendations need to consider this.

With this review we have found sufficient evidence to recommend antibiotic use for the reduction in mortality and morbidity due to *Shigella* and *Cryptosporidium* diarrhea. We have identified a need for further research in this field and recommend that more clinical trials should be conducted to evaluate efficacy and safety of first- and second- line drugs currently in use for treatment for diarrhea in both developing and developed countries to strengthen the evidence of the recommended antibiotics.

## Competing interests

The authors declare no conflict of interests.

## Authors' contributions

Dr ZAB was responsible for designing the review and co-ordinating the review. JKD, AA and RAS were responsible for: data collection, screening the search results, screening retrieved papers against inclusion criteria, appraising quality of papers, abstracting data from papers, entering data into RevMan, analysis and interpretation of data and writing the review. ZAB and JKD critically reviewed and modified the manuscript.

## Declarations

The publication costs for this supplement were funded by a grant from the Bill & Melinda Gates Foundation to the US Fund for UNICEF (grant 43386 to "Promote evidence-based decision making in designing maternal, neonatal, and child health interventions in low- and middle-income countries”). The Supplement Editor is the principal investigator and lead in the development of the Lives Saved Tool (LiST), supported by grant 43386. He declares that he has no competing interests.

This article has been published as part of *BMC Public Health* Volume 13 Supplement 3, 2013: The Lives Saved Tool in 2013: new capabilities and applications. The full contents of the supplement are available online at http://www.biomedcentral.com/bmcpublichealth/supplements/13/S3.

## References

[B1] UNICEF Levels & Trends in Child Mortality, Estimates Developed by the UN Inter-agency Group for Child Mortality Estimation 2012 New York, NY: UNICEF

[B2] Bhattacharya SK Datta D Bhattacharya MK Garg S Ramamurthy T Manna B Nair GB Nag A Moitra A Cholera in young children in an endemic area Lancet 1992 340 8834-8835 1549 1361637 10.1016/0140-6736(92)92804-o

[B3] Deen JL von Seidlein L Sur D Agtini M Lucas ME Lopez AL Kim DR Ali M Clemens JD The high burden of cholera in children: comparison of incidence from endemic areas in Asia and Africa PLoS Negl Trop Dis 2008 2 2 e173 10.1371/journal.pntd.0000173 18299707 PMC2254203

[B4] Glass RI Becker S Huq MI Stoll BJ Khan MU Merson MH Lee JV Black RE Endemic cholera in rural Bangladesh, 1966-1980 Am J Epidemiol 1982 116 6 959 970 7148820 10.1093/oxfordjournals.aje.a113498

[B5] Sack RB Siddique AK Longini IM Jr. Nizam A Yunus M Islam MS Morris JG Jr. Ali A Huq A Nair GB A 4-year study of the epidemiology of Vibrio cholerae in four rural areas of Bangladesh J Infect Dis 2003 187 1 96 101 10.1086/345865 12508151

[B6] World Health Organization Cholera, 2010 Weekly Epidemiological Record 2010 86 31 325 339

[B7] World Health Organization Cholera outbreak: assessing the outbreak response and improving preparedness 2004 WHO: Geneva, Global Task Force on Cholera Control Available at http://whqlibdoc.who.int/hq/2004/WHO_CDS_CPE_ZFk_2004.4_eng.pdf

[B8] Siddique AK Nasim SMA Guidelines for Operating Makeshift Treatment Centres in Cholera Epidemics 1997 ICDDR, B, Dhaka Available at https://centre.icddrb.org/images/epidemics.pdf

[B9] Nelson EJ Nelson DS Salam MA Sack DA Antibiotics for both moderate and severe cholera N Engl J Med 364 1 5 7 21142691 10.1056/NEJMp1013771

[B10] World Health Organization Guidelines for the control of shigellosis, including epidemics due to Shigella dysenteriae type 1 2005 WHO Document Production Services, Geneva, Switzerland Available at http://whqlibdoc.who.int/publications/2005/9241592330.pdf

[B11] Amieva MR Important bacterial gastrointestinal pathogens in children: a pathogenesis perspective Pediatr Clin North Am 2005 52 3 749 777 vi 10.1016/j.pcl.2005.03.002 15925661

[B12] Ram PK Crump JA Gupta SK Miller MA Mintz ED Part II. Analysis of data gaps pertaining to Shigella infections in low and medium human development index countries, 1984-2005 Epidemiol Infect 2008 136 5 577 603 17686195 10.1017/S0950268807009351 PMC2870860

[B13] Kotloff KL Winickoff JP Ivanoff B Clemens JD Swerdlow DL Sansonetti PJ Adak GK Levine MM Global burden of Shigella infections: implications for vaccine development and implementation of control strategies Bull World Health Organ 1999 77 8 651 666 10516787 PMC2557719

[B14] Bardhan P Faruque ASG Naheed A Sack DA Decreasing Shigellosis-related Deaths without Shigella spp.-specific Interventions, Asia Emerging Infectious Diseases 2010 16 11 1718 10.3201/eid1611.090934 21029529 PMC3294502

[B15] Clark DP New insights into human cryptosporidiosis Clin Microbiol Rev 1999 12 4 554 563 10515902 10.1128/cmr.12.4.554 PMC88924

[B16] Mosier DA Oberst RD Cryptosporidiosis. A global challenge Ann N Y Acad Sci 2000 916 102 111 11193609 10.1111/j.1749-6632.2000.tb05279.x

[B17] Berkman DS Lescano AG Gilman RH Lopez SL Black MM Effects of stunting, diarrhoeal disease, and parasitic infection during infancy on cognition in late childhood: a follow-up study Lancet 2002 359 9306 564 571 10.1016/S0140-6736(02)07744-9 11867110

[B18] Ochoa TJ Salazar-Lindo E Cleary TG Management of children with infection-associated persistent diarrhea Semin Pediatr Infect Dis 2004 15 4 229 236 10.1053/j.spid.2004.07.003 15494946

[B19] Smith HV Corcoran GD New drugs and treatment for cryptosporidiosis Curr Opin Infect Dis 2004 17 6 557 564 10.1097/00001432-200412000-00008 15640710

[B20] Christopher PRH David KV John SM Sankarapandian V Antibiotic therapy for Shigella dysentery The Cochrane Library 2009 19821387 10.1002/14651858.CD006784.pub2

[B21] Traa BS Walker CL Munos M Black RE Antibiotics for the treatment of dysentery in children Int J Epidemiol 2010 39 Suppl 1 i70 74 20348130 10.1093/ije/dyq024 PMC2845863

[B22] Walker N Fischer-Walker C Bryce J Bahl R Cousens S Standards for CHERG reviews of intervention effects on child survival International journal of epidemiology 2010 39 suppl 1 i21 i31 20348122 10.1093/ije/dyq036 PMC2845875

[B23] Kabir I Khan WA Haider R Mitra AK Alam AN Erythromycin and trimethoprim-sulphamethoxazole in the treatment of cholera in children J Diarrhoeal Dis Res 1996 14 4 243 247 9203786

[B24] Roy SK Islam A Ali R Islam KE Khan RA Ara SH Saifuddin NM Fuchs GJ A randomized clinical trial to compare the efficacy of erythromycin, ampicillin and tetracycline for the treatment of cholera in children Trans R Soc Trop Med Hyg 1998 92 4 460 462 10.1016/S0035-9203(98)91094-X 9850410

[B25] Zimbabwe, Bangladesh, South Africa (Zimbasa) Dysentery Study Group Multicenter, randomized, double blind clinical trial of short course versus standard course oral ciprofloxacin for Shigella dysenteriae type 1 dysentery in children Pediatr Infect Dis J 2002 21 12 1136 1141 12488664 10.1097/00006454-200212000-00010

[B26] Alam AN Islam MR Hossain MS Mahalanabis D Hye HK Comparison of pivmecillinam and nalidixic acid in the treatment of acute shigellosis in children Scand J Gastroenterol 1994 29 4 313 317 10.3109/00365529409094842 8047805

[B27] Salam MA Dhar U Khan WA Bennish ML Randomised comparison of ciprofloxacin suspension and pivmecillinam for childhood shigellosis Lancet 1998 352 9127 522 527 10.1016/S0140-6736(97)11457-X 9716056

[B28] Varsano I Eidlitz-Marcus T Nussinovitch M Elian I Comparative efficacy of ceftriaxone and ampicillin for treatment of severe shigellosis in children J Pediatr 1991 118 4 Pt 1 627 632 2007941 10.1016/s0022-3476(05)83392-x

[B29] Haltalin KC Nelson JD Kusmiesz HT Comparative efficacy of nalidixic acid and ampicillin for severe shigellosis Arch Dis Child 1973 48 4 305 312 10.1136/adc.48.4.305 4574641 PMC1648332

[B30] Amadi B Mwiya M Musuku J Watuka A Sianongo S Ayoub A Kelly P Effect of nitazoxanide on morbidity and mortality in Zambian children with cryptosporidiosis: a randomised controlled trial Lancet 2002 360 9343 1375 1380 10.1016/S0140-6736(02)11401-2 12423984

[B31] Rossignol JF Ayoub A Ayers MS Treatment of diarrhea caused by Cryptosporidium parvum: a prospective randomized, double-blind, placebo-controlled study of Nitazoxanide J Infect Dis 2001 184 1 103 106 10.1086/321008 11398117

[B32] Wittenberg DF Miller NM van den Ende J Spiramycin is not effective in treating cryptosporidium diarrhea in infants: results of a double-blind randomized trial J Infect Dis 1989 159 1 131 132 10.1093/infdis/159.1.131 2642518

[B33] Sack DA Sack RB Nair GB Siddique AK Cholera Lancet 2004 363 9404 223 233 10.1016/S0140-6736(03)15328-7 14738797

[B34] Saha D Karim MM Khan WA Ahmed S Salam MA Bennish ML Single-dose azithromycin for the treatment of cholera in adults New England Journal of Medicine 2006 354 23 2452 2462 10.1056/NEJMoa054493 16760445

[B35] Nelson EJ Nelson DS Salam MA Sack DA Antibiotics for both moderate and severe cholera New England Journal of Medicine 2011 364 1 5 7 10.1056/NEJMp1013771 21142691

[B36] Snelling WJ Xiao L Ortega-Pierres G Lowery CJ Moore JE Rao JR Smyth S Millar BC Rooney PJ Matsuda M Cryptosporidiosis in developing countries J Infect Dev Ctries 2007 1 3 242 256 19734601

[B37] Hunter PR Nichols G Epidemiology and clinical features of Cryptosporidium infection in immunocompromised patients Clin Microbiol Rev 2002 15 1 145 154 10.1128/CMR.15.1.145-154.2002 11781272 PMC118064

